# Quantifying cell adhesion through forces generated by acoustic streaming

**DOI:** 10.1016/j.ultsonch.2022.106204

**Published:** 2022-10-13

**Authors:** Chikahiro Imashiro, Jiyang Mei, James Friend, Kenjiro Takemura

**Affiliations:** a Institute of Advanced Biomedical Engineering and Science, Tokyo Women’s Medical University, 8-1 Kawada-cho, Shinjuku-ku, Tokyo 162-8666, Japan; b Department of Mechanical Engineering, Keio University, 3-14-1 Hiyoshi, Kohoku-ku, Yokohama, Kanagawa 223-8522, Japan; c Medically Advanced Devices Laboratory, Center for Medical Devices, Department of Mechanical and Aerospace Engineering, Jacobs School of Engineering and Department of Surgery, School of Medicine, University of California, San Diego, CA 92093, USA

**Keywords:** Cell adhesion, Cell detachment, Lamb wave, Acoustic streaming, Lab on a chip

## Abstract

The strength of cell adhesion is important in understanding the cell’s health and in culturing them. Quantitative measurement of cell adhesion strength is a significant challenge in bioengineering research. For this, the present study describes a system that can measure cell adhesion strength using acoustic streaming induced by Lamb waves. Cells are cultured on an ultrasound transducer using a range of preculture and incubation times with phosphate-buffered saline (PBS) just before the measurement. Acoustic streaming is then induced using several Lamb wave intensities, exposing the cells to shear flows and eventually detaching them. By relying upon a median detachment rate of 50 %, the corresponding detachment force, or force of cell adhesion, was determined to be on the order of several nN, consistent with previous reports. The stronger the induced shear flow, the more cells were detached. Further, we employed a preculture time of 8 to 24 h and a PBS incubation time of 0 to 60 min, producing cell adhesion forces that varied from 1.2 to 13 nN. Hence, the developed system can quantify cell adhesion strength over a wide range, possibly offering a fundamental tool for cell-based bioengineering.

## Highlights

• Cell adhesion strength was quantified using acoustic streaming.
• Lamb wave gives cells regulated shear flow in our device.
• The stronger the induced shear flow is, the more cells were detached.
• Cell adhesion strength was affected by culture conditions.
• The validity of a developed device was supported by previous reports.

## 1 Introduction

The many applications that define bioengineering—the development of drugs or biomaterials, tissue engineering, embryology, and fundamental biology—are predicated upon controlling and understanding cellular function and behavior. The adhesion of cells is key to defining their behavior in both the *in vitro* and *in vivo* environments typical in bioengineering, and thus there have been many studies of cell adhesion reported in the literature [1]. In the *in vivo* context, the evaluation of cell adhesion aids understanding of disease and in developing novel therapies to overcome them. A famous example is the weaker adhesion of cancerous cells, producing greater shedding of cells from tumors into the blood: metastasis [2], [3]. Increasing the adhesion of cancer cells is one route to reducing metastasis as a therapeutic method [4] as another interesting and recent method.

Cell adhesion is also important *in vitro*. Cells are often cultured and harvested, released from adherent surfaces to either be used or seeded again onto a surface to increase the number of cells. Enzymes are used in conventional cell culture protocols to release the cells during harvesting, though these enzymes reduce the cells’ functionality such as proliferation rate or viability due to the digestion of proteins [5], [6], [7], [8], [9], [10]. Additionally, there are many studies reporting the improvement of cell adhesion on novel materials to produce new biomaterials or to generate cell aggregates for tissue engineering [11], [12]. Despite such attention, there are few technologies to measure cell adhesion, and none known to the authors that are broadly useful or provide adhesion measurements without contact.

There are two approaches to measuring cell adhesion. One approach considers individual cells, and the other considers populations of cells [1]. Individual cells of identical type and conditions will exhibit significantly different adhesion, because the morphology of the cells can be quite different [13]. For this reason, and to produce results hopefully useful for more generally understanding the adherent behavior of cells, we focus on cell populations to determine the overall distribution of adhesion strength. Centrifugal and flow-driven shear force methods are the principal means used to measure the adhesion of populations of cells [14], [15], [16]. A key advantage of using flow-driven shear is the ability to observe the cells during detachment. The centrifugal method spins the sample at high speed, typically preventing detailed views of the cells. In the flow-driven shear method, the fluid surrounding the cells is made to flow in a particular direction, producing shear upon the adherent cells exposed to it. The flow rate in this method is both important and difficult to control [17], reducing the precision of experiments that use the method. It also can affect the cells’ function in an uncontrolled way via mechanotransduction [18], [19], which is never desirable. Consequently, the evaluation of the cell adhesion for a population of cells is troublesome. We thus need to develop a cell adhesion measurement protocol where a properly regulated shear flow can be produced upon a cultured cell population.

**Fig. 1:**
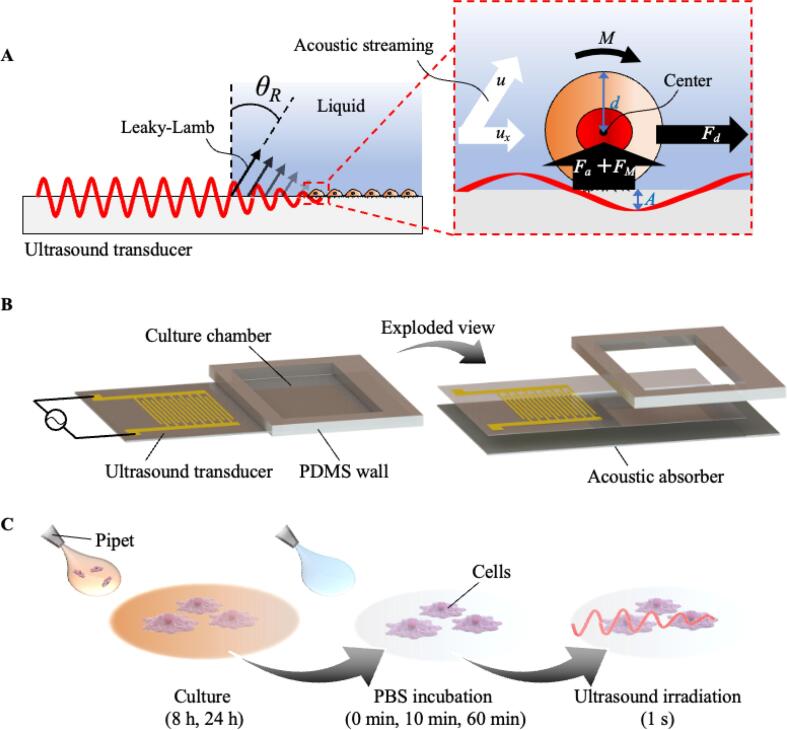
(Color online). Schematic of the cell strain and detachment system. A: Mechanical stimulus on cells. Note that the size of culture chamber is 17 mm × 9 mm. B: The developed device. C: Procedures performed for the experiment in this study.

With this in mind, we developed a method to determine the adhesion force present on cells using Lamb wave-generated acoustic streaming [20], [21], [22]. Acoustic streaming rapidly generates fluid flow and provides precise control of the flow by controlling the input signal into the MHz-range ultrasound transducer; this flow is passed over adherent cells as shown in Fig. 1. Mouse myoblasts (C2C12) were used in this study as a typical adhesive cell species. Further, the incubation time with phosphate-buffered saline (PBS) and the culture time were employed as factors to regulate the cell adhesion strength, and as a result the adhesive strength was changed, consistent with past results [23]. In the results that follow, we demonstrate the ability to quantify the adhesion force of adherent cells through acoustic streaming-driven fluid shear.

## 2 Theory and system design

### 2.1 System design inducing acoustic streaming

When a liquid is in contact with an ultrasound transducer, Lamb wave propagating on the substrate induces acoustic streaming in the liquid at the Rayleigh angle, *θ_R_*, which is determined by the difference in the phase velocity of the Lamb wave in the substrate and the acoustic wave it generates in the fluid (Fig. 1A) [24]. We developed a system in which cells can be cultured directly upon the ultrasound transducer, which accurately regulates acoustic streaming as shown in Fig. 1B. A 128° Y-rotated X-propagating lithium niobate (LN, Precision Micro-Optics Inc., Burlington, MA, USA) substrate with an interdigital transducer was used as the ultrasound transducer. The design of the interdigital transducer is shown in Fig. S1; it was designed to form acoustic waves at a frequency of 10 MHz. At 10 MHz, the IDT will form a Lamb wave. Any spurious bulk waves were prevented by using an acoustic absorber (Scotch® Removable Adhesive Putty Thermo, 3 M Company, Saint Paul, MN, USA) on the bottom side of the lithium niobate, which left the Lamb wave mostly unaffected. While higher frequencies might be useful and could be used to generate surface acoustic waves at above 40 MHz in a 500-µm thick substrate, for example, we chose this low frequency to avoid driving atomization from the fluid interface of the cell media [25]. At this low frequency, the attenuation of the Rayleigh wave into the substrate due to the difference in wave speed between the surface and the bulk of the piezoelectric media is insufficient, allowing the wave to penetrate all the way through to the other side of the substrate. This type of wave is a Lamb wave, with otherwise identical propagation speed and wavelength to the Rayleigh (SAW) wave. Lamb waves have been reported in the literature to produce useful acoustofluidics phenomena [26]. Bulk waves can also be formed, though the 127.68°Y-rotated cut of LN was originally chosen to help suppress such waves, and in our case the presence of the lossy mount below the LN will eliminate them. Though the Lamb wave also produces motion on the bottom face of the LN, and therefore also loses energy to its lossy mounting below, it is generated from the entire bulk of the LN beneath the IDT and is therefore sufficiently powerful to still drive acoustic streaming and eventual cell detachment despite the loss. Notably, bulk waves, and, for that matter, flexural waves will have much shorter wavelengths, and thus the region over which these waves are generated by the IDT through piezoelectric coupling is much smaller than the Lamb wave. Consequently, these other waves are much weaker—and also absent, as we observed only waves at the wavelength of the Lamb wave on the substrate in our system. While it may be suggested that the Lamb wave in our system is a lossy Lamb wave akin to a lossy Rayleigh wave, all Lamb waves are lossy in this way, and so we just call them Lamb waves here. To form a cell culture chamber, polydimethylsiloxane (PDMS) wall was attached onto the ultrasound transducer by surface tension. A function generator (WF1946B, NF Corp., Kanagawa, Japan) and an amplifier (LZY‐22+; Mini‐Circuits; Brooklyn, NY USA) were used to input an RF signal for driving the ultrasound transducer. The observation area for cell adhesion evaluation was limited to the region shown in Fig. S2.

### 2.2 Cell detachment mechanism

Generally speaking, in flow a small object that adheres to a surface should experience hydrodynamic drag, torque, and lift, all which contribute to removing the object [27]. Among these factors, the lifting force can be always ignored with microparticles [27]. In our system, thus, the possible forces responsible for cell detachment include a drag force upon the cells from acoustic streaming, *F_d_*; a vertical force on the cells, *F_M_,* due to a torque, *M*, also from the acoustic streaming; and an acoustic radiation force, *F_a_*, as shown in Fig. 1A. The force, *F_d_* is given by.

(1) Fd=3πΓρffd2ux22C1+0.15Re0.678,

where*Γ*, $\rho_{f}$, *f*, *d*, $u_{x}$, *C* and *Re*, respectively represent the linear profile in the viscous sublayer (=1), the density of PBS (=1.007 × 10^3^ kg/m^3^), a correction factor for the presence of the wall (the substrate the cell is attached to, =1.7009), the cell diameter (=10 µm), the shear velocity, the Cunningham correction factor (≈ 1), and the Reynolds number [28]. Note that since in our set up PBS should be a continuum, the Cunningham correction factor of 1 is used. The moment *M* is given by.

(2) $$M=\frac{2\pi \mu f_{m}d^{2}u_{x}}{C},$$

where μ and *f_m_* represent the viscosity coefficient of PBS (=8.94 × 10^-4^ Pa·s) and the wall effect correction factor (=0.943993) [29]. Further, when the diameter of cell adhesion area is assumed to be *d*, the vertical force, *F_M_*, is given by.

(3) $$F_{M}=\frac{2M}{d}$$

When a standing wave is generated in the fluid cavity, the acoustic radiation force, *F_a_*, produced upon objects present in the cavity is given by [30].

(4) $$F_{a}=\frac{1}{3}\pi^{2}\rho {\left|A\right|}^{2}\kappa^{3}{\left(\frac{d}{2}\right)}^{5}\frac{5-2\frac{\rho_{c}}{\rho_{f}}}{2+\frac{\rho_{c}}{\rho_{f}}}\sin\left(2\kappa d\right),$$

where $\left|A\right|$, κ, and $\rho_{c}$ represent the particle velocity, the wavenumber, and the density of cells (=1.08 × 10^3^ kg/m^3^) [31], respectively. Note that we assumed that the diameter of the cell’s adhesion area is the same as the cell diameter and that the physical properties of the cell used for the numerical analysis are for a different cell line derived from mammalians.

## 3 Results

### 3.1 Evaluating mechanical stimulation on cells

Next, we evaluated the effect of producing shear upon the cells. Note that the effective driving frequency for the ultrasound transducer was identified by measuring the electrical admittance; we chose a local maximum at 9.62 MHz (Fig. S4) for every experiment as our operating frequency. The true resonance frequency is often slightly less than the design resonance frequency in interdigital electrodes due to the weight of the metal of the electrodes upon the substrate.

**Fig. 2:**
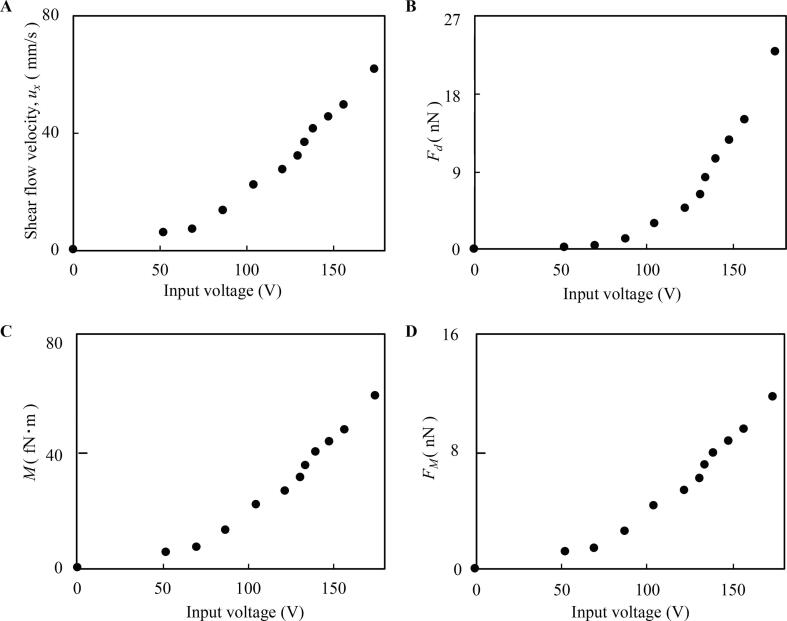
Evaluating drive performance of the cell detaching device. Shear flow velocity, *ux* (A), a drag force on the adhesive position of the cell from acoustic streaming, *Fd* (B), torque on the cell from acoustic streaming, *M* (C), and vertical force on the cell due to *M, F_M_* (D) were measured and calculated.

As shown in Fig. 2A, the relationship between the input voltage and the flow rate of induced acoustic streaming was investigated by particle image velocimetry (PIV). Note that the region of interest and quiver plots used for evaluating acoustic streaming are shown in Figs. S2 and S3, respectively. It is shown that uniform and stable flow can be generated in the developed device. Based on this result, *F_d_*, *M*, and *F_M_* were calculated as shown in Fig. 2B, C, and D, respectively, using equations (1–3). Here, we have also calculated the acoustic radiation force, *F_a_*, based on the vibrational displacement of the culture surface while using an input voltage of 173.98 V, the maximum amplitude we could generate. Note that the relationship between the input voltage and the maximum vibrational displacement is shown in Fig. S5. Since the displacement was calculated to be 0.5 nm by a finite element analysis software (COMSOL Multiphysics ver. 5.5, COMSOL Inc., Palo Alto, CA. See Supplemental Note 1), and the acoustic velocity of PBS was assumed to be 1500 m/s, we were able to estimate *F_a_* to be 1.7 × 10^-7^ nN at most, negligible compared to *F_d_* and *F_M_* shown in Fig. 2B and D.

### 3.2 The viability of the cells exposed to this system

The viability of the cells exposed to this system was assessed via two aspects: the characteristics of the growth culture during cell culture, and the cells themselves after detachment.

**Fig. 3:**
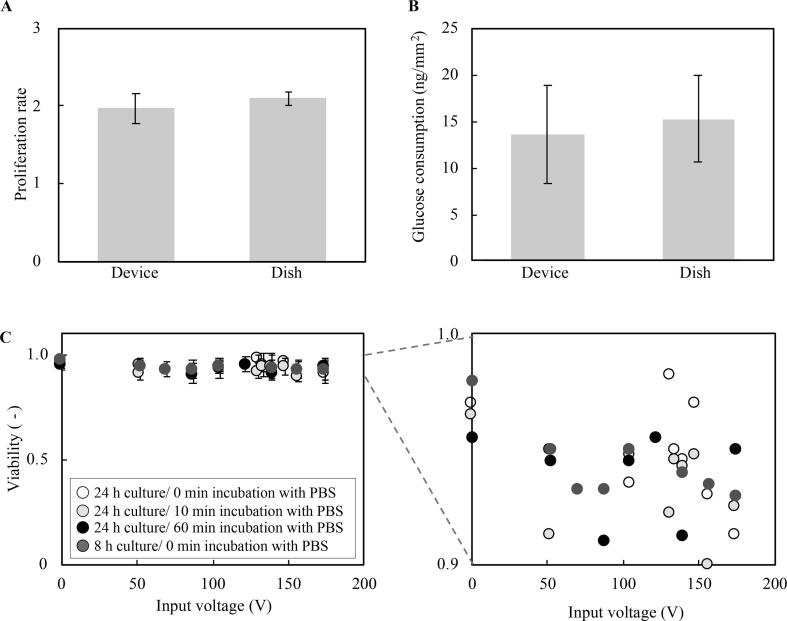
Biocompatibility of the system. A: The cell proliferation rate is not significantly affected by the use of the ultrasound transducer in comparison to the control culture dish (mean ± SD, *n* = 4, student *t*-test). B: The glucose consumption of the cells is unaffected by use of the ultrasound transducer compared to the culture dish control (mean ± SD, *n* = 4, student *t*-test). C: The viability of the cells is not significantly affected by the use of different culture or PBS incubation conditions, or the use of different input voltages, indicating the ultrasound transducer does not affect the cells’ viability (mean ± SD, *n* = 4). Detail of the data in the left plot is enlarged in the right figure to indicate the absence of any trend. Sample sizes indicated are biological replicates.

To evaluate the biocompatibility of the developed device, C2C12 cells with a density of 1.0 × 10^2^/mm^2^ in culture media were seeded into the acoustic device chamber, with a conventional, 35-mm diameter culture dish as a control. Cell proliferation and glucose consumption were then measured for 24 h during culture as shown in Fig. 3A and B using a cell counter (1450101 J1, Bio Rad, Hercules, CA, USA) and glucose assay kit (GAHK-20, Sigma-Aldrich, MO, USA) according to their protocol, respectively. There were no statistically significant differences between the results for the acoustic device chamber and the control culture dish, which indicates good biocompatibility for the acoustic device.

The viability of the detached cells was evaluated after cell detachment experiments with several conditions as shown in Fig. 3C. Most of the cells were detached and remained viable after the experiment. It was also shown that there is no trend among each experimental condition, as shown in Fig. 3C. A live dead assay and the evaluation of the cell proliferation were performed using trypan blue staining (Trypan Blue Solution; Thermo Fisher Scientific, Waltham, MA, U.S.) and an automatic cell counter (1450101 J1, Bio-Rad, Hercules, CA, USA).

### 3.3 Cell detachment ratio increases with input voltage

**Fig. 4:**
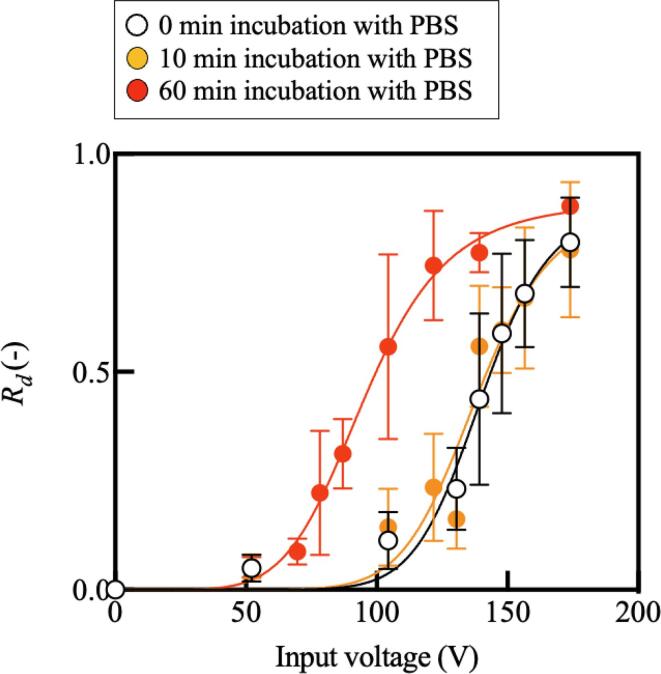
(Color online). The result of the cell detachment experiment with different incubation times with PBS (mean ± SD, *n* = 4). Note that cells were cultured for 24 h before the experiments. We fit sigmoid functions to the data plot with 0, 10, and 60 min incubation with PBS, setting the bottom at 0. They have half maximal inhibitory concentrations (IC50) of 140.4, 138.2, and 95.8 V and Hill slope of 10.5, 9.5, and 6.0, respectively. Sample size indicated is biological replicates.

We next used the system to assess cell adhesion after four distinctly different culture conditions. Initially, 1.0 × 10^3^ C2C12 cells were seeded in the culture chamber and then cultured for either 8 or 24 h. After this time, and immediately prior to using the ultrasound transducer to produce shear upon the cells, the culture media was removed. Afterwards, 153 µL of PBS was introduced into the chamber. The ultrasound transducer was then driven at a defined input voltage from zero to 173.98 V for 1 s, detaching a fraction of the total number of adherent cells, defined as the *cell detachment ratio*, *R_d_*, and plotted in Fig. 4 as a function of the input voltage. As shown in this figure, *R_d_* monotonically increases with the input voltage.

### 3.4 Cell adhesion weakens due to immersion in PBS

The relationship between the cell adhesion and the time the cells were incubated in PBS was then investigated. Approximately 1.0 × 10^3^ C2C12 cells were seeded and cultured in culture media for 24 h before the cell detachment experiment. The culture media was then removed, and 153 µL of PBS.

was introduced. The cells were immersed in PBS for 10 and 60 min before cell detachment. The ultrasound transducer was then driven for 1 s at each voltage. The relationship between the input voltage and the cell detachment ratio, *R_d_*, shows a monotonically increasing trend in Fig. 4. Further, we defined a threshold voltage for cell detachment, *V_d_*, at which half of the cells were detached from the substrate (*R_d_* = 0.5)— the half maximum inhibitory concentration (IC50). The value of *V_d_* was determined by approximating the cell detachment as a logistic process, and then defining *V_d_* at the midpoint of this process. As a result, *V_d_* decreased with an increase in the incubation time with PBS. After incubating for 0, 10, and 60 min in PBS, *V_d_* was found to be 140.4, 138.2, and 95.8 V, respectively. Further, the maximum slope of each logistic curve was 10.5, 9.5, and 6.0, respectively.

### 3.5 Cell adhesion getting strengthened during culture

**Fig. 5:**
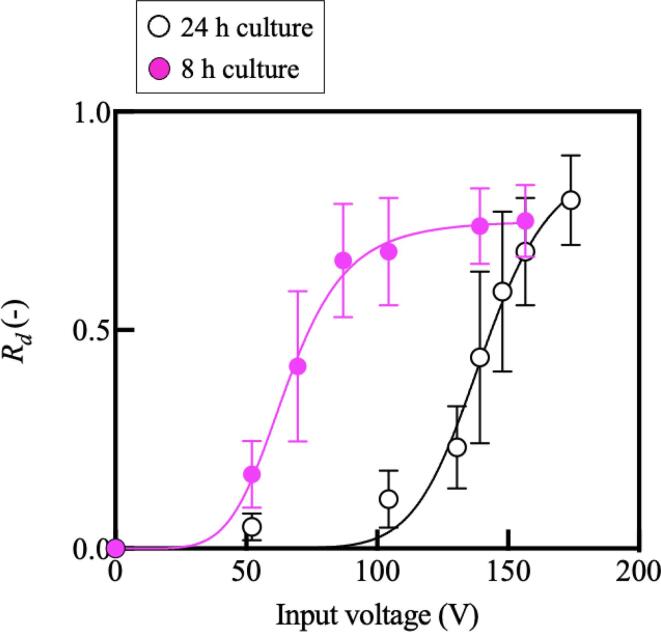
(Color online). The result of the cell detachment experiment with different culture times (mean ± SD, *n* = 4). Note that the incubation with PBS was not employed here. We fit sigmoid functions to the data plot after 8 and 24 h culture, noting that a zero voltage produces zero detachment. They have half maximal inhibitory concentrations (IC50) of 65.6 and 140.4 V and a maximum slope of 5.7 and 10.5, respectively. Sample size indicated is biological replicates.

To evaluate the relationship between the cell culture time and the cell adhesion, 1.0 × 10^3^ C2C12 cells were seeded and then cultured for 8 h before the cell detachment experiment. Cells then underwent a cell detachment experiment with exposure to the Lamb wave at different input v oltages right after the replacement of culture media with PBS. Fig. 5 shows the result of the cell detachment experiment comparing the effect of culture times. White plots representing the result of the cells cultured for 24 h are provided again for comparison. As shown, *V_d_* decreased with a shorter culture time, indicating cell adhesion strengthens during culture. Note that *V_d_* after 8- and 24-h cultures were 65.6 and 140.4 V, respectively. Further, the maximum slope of the logistic approximation of the detachment was 5.7 and 10.5, respectively.

## 4 Discussion

We present a system that can quantitatively evaluate cell adhesion strength using acoustic streaming for detaching cells. In this system, cells are cultured on the ultrasound transducer, which enables a stable regulation of shear flow applied to cells as shown in Fig. 2. Note that, the immediate and stable generation of acoustic streaming by an ultrasound transducer has been reported in previous research [32], [33], [34]. On the other hand, viability of cells is a concern; however, Fig. 3 shows the biocompatibility of the developed system. Cells are exposed to acoustic streaming-induced shear flow and the acoustic radiation force at once. The latter is, however, calculated to be negligible compared to the former, which indicates that cell adhesion capability can be evaluated by properly regulated acoustic streaming-driven shear flow. The result of our cell detachment experiments shows a monotonically increasing trend of detachment ratio with a growing shear flow as shown in Fig. 4, Fig. 5. The trend is consistent with previous research [35]. Further, experiments were performed to evaluate the cell adhesion with different culture and PBS immersion times. It was demonstrated that the cell adhesion capability is affected by the culture condition of the cells, also consistent with previous research [35]. The most important contribution of this study is that the effects of culture conditions on the cell adhesion strength can now be quantitatively evaluated. To measure the certain cell’s adhesion force individually, other technologies based on fine probes should be better[36]. However, our method has a strong point capable of grabbing the effect of a culture environment briefly since averaged cell adhesion strength can be measured at once. Further, since the cell detaching methods utilizing acoustic force and acoustic streaming have gotten attention [37], [38], this study can offer an indication to decide the condition of cell detachment. The discussion about the validity and reliability of the developed system and the acquired results are detailed below.

The key point of this study is the employment of Lamb waves as a driving force generating shear flow, which may be a factor for cell detachment [39], [40]. There might be several concerns we have to take into account for employing Lamb waves as a potential method to detach cells: acoustic radiation force, deformation, diffusion of chemical species, and heat generation. The acoustic radiation force is a negligible factor as shown. In this study, we assumed the standing wave in the liquid, which produces a stronger acoustic force compared to traveling acoustic waves. Even with a standing wave, the acoustic force is small enough to be neglected compared to shear flow driven by streaming. Regarding the cell deformation, the wavelength of sound wave is about 156 µm, determined knowing the sound speed in the media is about 1500 m/s and the frequency is 9.62 MHz. The wavelength is therefore much larger than the cellular diameter of 10 µm. Hence, the cells are exposed to an essentially homogeneous tensile force [41]. Further, the vibration amplitude is at most 0.5 nm, and so the cells are exposed to 0.005 % stretch, which should be negligible compared with the previous studies applying several tens of percent stretch to examine mechanotransduction [42], [43]. Since such a large deformation was required to regulate cell function without driving cell detachment in the previous research, we argue that the deformation induced by direct acoustic forces in our experiment itself is negligible for detaching cells [44]. As for the diffusion of chemical species, if any chemical species to detach cells such as enzymes were applied, we should take the possibility into account [8]. However, PBS was utilized to regulate cell adhesion in this study, which indicates such diffusion should be irrelevant. Although heat generation due to ultrasound propagation is an important concern, temperature variation cannot be measured in our system due to the short exposure time of the ultrasound. There is, however, active convection due to acoustic streaming, which disperses generated heat and helps to prevent any temperature increase. Further, in this study, the temperature increase is not supposed to contribute to cell detachment. When cells are detached by an increased temperature, the cause should be necrosis or regulated protein expression. However, the number of dead cells was not observed to increase in the experiments, and the ultrasound exposure duration of 1 s is too short to regulate the protein expression [45], [46]. Further, even with temperature stimulation inducing apoptosis, cells were not detached in previous research with such a short culture time [47]. Notably, the effect of electric field transport with the acoustic wave across the bare LN substrate that is important at the nano scale [48] and used for other purposes [49] has no observable effect on detaching adherent cells. We, thus, concluded this device can evaluate cell adhesion strength solely using shear flow induced by ultrasound-driven acoustic streaming.

In our system, cells are directly cultured on the ultrasound transducer and exposed to ultrasound irradiation, which offers both advantages and disadvantages. Lamb wave propagated on the surface of an ultrasound transducer can be precisely regulated [50]. The driving frequency of the Lamb wave in the substrate is determined by the design of the interdigital transducer. This helps narrowly regulate the operating frequency, together with the weak dependence of the single crystal lithium niobate substrate on temperature and the fact that it is not hysteretic [51], such that it does not directly generate heat. Further, to effectively generate acoustic streaming, using rapidly attenuating high-frequency ultrasound is an important requirement. ultrasound transducers having IDTs are ideal for this purpose. While the advantages of the device were shown, a legitimate concern in using the ultrasound transducer as a culture surface may be raised: the evaluation of cell adhesion onto such a special substrate. Biocompatibility is not a concern, as several researchers have cultured cells on LN, the main material of the ultrasound transducers, without observation of cytotoxicity [52]. Further, the proliferation rate and glucose consumption of the cells, which are measures of biocompatibility, were not affected by the culture on LN as shown in Fig. 3C. In any case, two separate studies have each indicated that mechanical stimulation of cells cultured on either LN or glass produce similar effects on the cells regardless of the culture surface [52], [53]. We conclude this method of evaluating the cell adhesion on the ultrasound transducer should be meaningful for cells bound onto glass substrates.

The cell adhesion strengths appear to be effectively described by the shear flow over a range of culture conditions in this study. Fig. 4, Fig. 5 show that the stronger the induced shear flow, the more cells are detached, which is qualitatively reasonable. Further, increasing the incubation time with PBS and decreasing the cell culture time weakens cell adhesion. Since cell adhesion is promoted by Mg^2+^, cell adhesion is weakened by removing Mg^2+^ from the culture medium [54]. In previous research, it has been reported that a longer culture with a solution containing no Mg^2+^ lowers cell adhesion [55]. Since the PBS used in this study does not contain Mg^2+^, the result shown in Fig. 4 is reasonable. Regarding the effect of culture time on cell adhesion, it has been reported that cell adhesion is strengthened with time *in vitro* [1], [56], and such a trend has also been shown in Fig. 5. Corresponding to the threshold voltage, *V_d_* to deliver a detachment ratio *R_d_* of 0.5, the threshold forces for horizontal/vertical directions are estimated as shown in Table 1 by using the results shown in Fig. 2. Further, the resultant adhesion forces are listed as a magnitude of adhesion force; *M_fa_*, and the ratio of *F_M_/F_d_* was also calculated. As shown, cells are detached with a force of 1.2–13 nN, corresponding to previous research reporting that the required force is from around 1 nN to around 8 nN [57]. In addition to the estimation of the adhesion force, an interesting trend was also found when focusing on the relationship between the adhesion force and the time of PBS incubation. The estimated cell adhesion forces, *F_M_*, *F_d_*, and *M_fa_*, were plotted with respect to the PBS immersion time (see Figs. S6) and found to trend downward with respect to PBS incubation time. Furthermore, we found that with an increase in PBS incubation time, both the applied voltage *V_d_* at which one half the cells were detached and the maximum slope of the logistic fit of the cells’ detachment decreased. This indicates that a shorter culture time and a longer incubation time with PBS increases the variance of the cell adhesion force. Since the cells’ adhesion force is reasonably expected to have a Gaussian distribution [58], this result is reasonable. The developed system offers a quantification methodology to evaluate cell adhesion strength under different culture conditions. For the estimation of the force on the cells, the simplified particle detachment model is used in this study, in which a plain surface and a spherical cell are assumed. This assumption produces a wall correction factor of 1.7009 [59]. However, other cells adhered to the surface may affect the flow induced by the Lamb wave, and potentially cells are not spherical. This simple modeling may be a potential confounding factor for the calculation of forces; however, as the results imply, the model provides an effective comparison of cell adhesion capabilities under different culture conditions. The development of a coefficient considering each cell shape should be required in future work for the accurate estimation of the cell adhesion strength. Calculating the force using finite element analysis may be a good candidate once we could obtain physical properties of cells. Further, the physical properties of different cell species and modeled shapes were used for numerical analysis in this study, which might cause a numerical error. Nevertheless, since we used the physical properties of mammalian cells same as C2C12 used in our study, the estimation should not be senseless. In the future direction of work, we could employ the reported techniques for the evaluation of a cell property to evaluate cell adhesion more accurately [60]. Nevertheless, since we used the physical properties of mammalian cells as C2C12 used in our study, the estimation should not be senseless. It may then be indicated that our study produces a significant impact on bioengineering by offering a quantified measurement of cell adhesion without need for contact.

**Table 1:** Estimated forces to detach the cells with each incubation time with PBS.

Culture time with media	24 h	24 h	24 h	8 h
Incubation time with PBS	0 min	10 min	60 min	0 min
Threshold voltage (*V_d_*)	140.4 V	138.2 V	95.8 V	65.6 V
Horizontal force (*F_d_*)	10.3 nN	9.7 nN	1.8 nN	0.3 nN
Vertical force (*F_M_*)	8 nN	7.5 nN	3.4 nN	1.2 nN
Magnitude of adhesion force (*M_af_*)	13.0 nN	12.3 nN	3.8 nN	1.2 nN
F_M_/F_d_	0.77	0.77	1.9	4.0

## 5 Conclusions

We developed a cell adhesion quantification method using acoustic streaming, and the validity and value of the developed system have been demonstrated. Interestingly, we revealed that when cells are exposed to even the same culture environment, the effect on cell adhesion can be diverse, the diversity of which is increased as the cell adhesion is reduced. Further, the results were supported by past publications, helping to validate our approach. In future work, the core idea of the developed system in employing high-frequency-ultrasound-induced acoustic streaming may be applied to different culture surfaces or even to the in/ex vivo environment, since we have already developed the technology to propagate ultrasound from the transducers to other materials [53], [61]. Cell adhesion can be evaluated in other conditions: other cell species, surface modifications engineered with mechanical or biochemical technologies, or culture environments with various chemical species or mechanical stimulation, all based on the idea of using acoustic streaming. We believe our study provides a fundamental technology to evaluate cell adhesion, and bioengineering research may be accelerated from this result.

## 6 Materials and methods

### 6.1 PIV analysis

The velocity of the shear flow was measured by PIV. For the measurement, the condition for the cell detachment experiment was replicated. Particles (19096–2, Polysciences, Inc., Warrington, England) with a diameter of 10 µm, similar to the diameter of cells, were used as tracers. Since the size of particles were smaller than the threshold size required for direct acoustic forces to be significant, the particles speed can be assumed as flow speed [62]. The motion of the particles was recorded at 2000 frames per second by high-speed cameras (VW-100C, VW-6000, and VH-Z50L; Keyence, Osaka, Japan). The field of view of the recording was set in the area shown in Fig. S2. PIV analysis was performed with a GUI-based open-source tool [PIVlab (version 2.38)].

### 6.2 Cell preparation

Mouse myoblasts (C2C12) (RCB0987; Riken Bio Resource Center, Ibaraki, Japan) were used in this study. Cells were cultured in growth medium [Dulbecco’s modified Eagle’s medium/F12 (Sigma-Aldrich, St. Louis, MO, USA) supplemented with 10 % fetal bovine serum (2917254; MP Biomedicals, Santa Ana, CA, USA)] in a 5 % CO2 humidified atmosphere incubator at 37 °C. Cell passaging was performed by trypsinization in 0.05 % trypsin-EDTA (25300; Life Technologies, Carlsbad, CA, USA).

### CRediT authorship contribution statement

**Chikahiro Imashiro:** Conceptualization, Data curation, Formal analysis, Funding acquisition, Investigation, Methodology, Project administration, Supervision, Visualization, Writing – original draft, Writing – review & editing. **Jiyang Mei:** Investigation, Methodology. **James Friend:** Data curation, Formal analysis, Funding acquisition, Resources, Supervision, Validation, Writing – review & editing. **Kenjiro Takemura:** Conceptualization, Data curation, Funding acquisition, Project administration, Resources, Validation, Visualization, Writing – review & editing.

## Declaration of Competing Interest

The authors declare the following financial interests/personal relationships which may be considered as potential competing interests: Chikahiro Imashiro reports financial support was provided by Japan Society for the Promotion of Science. Kenjiro Takemura reports financial support was provided by Japan Society for the Promotion of Science. James Friend reports financial support was provided by the W.M. Keck Foundation.

## Data Availability

Data will be made available on request.
