# Supplementary material for: Quantifying cell adhesion through forces generated by acoustic streaming
Source: Ultrason Sonochem. 2022 Oct 13;90:106204. doi: 10.1016/j.ultsonch.2022.106204 (PMC9583098; doi:10.1016/j.ultsonch.2022.106204)
Supplement: Supplementary data 1 [file mmc1.pdf]

Quantifying cell adhesion through forces generated by acoustic streaming

Chikahiro Imashiro,[1,2] Jiyang Mei,[3] James Friend,[3] and Kenjiro Takemura[2]

[1] Institute of Advanced Biomedical Engineering and Science, Tokyo Women's Medical University, 8-1 Kawada-cho, Shinjuku-ku, Tokyo 162-8666, Japan

[2] Department of Mechanical Engineering, Keio University, 3-14-1 Hiyoshi, Kohoku-ku, Yokohama, Kanagawa 223-8522, Japan

[3] Medically Advanced Devices Laboratory, Center for Medical Devices, Department of Mechanical and Aerospace Engineering, Jacobs School of Engineering and Department of Surgery, School of Medicine, University of California San Diego, CA 92093, USA

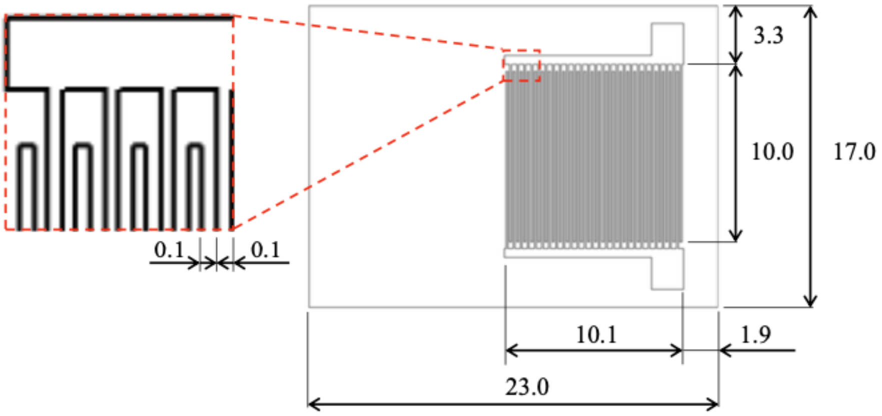

Figure S1 The design of the interdigital transducer. Unit: mm

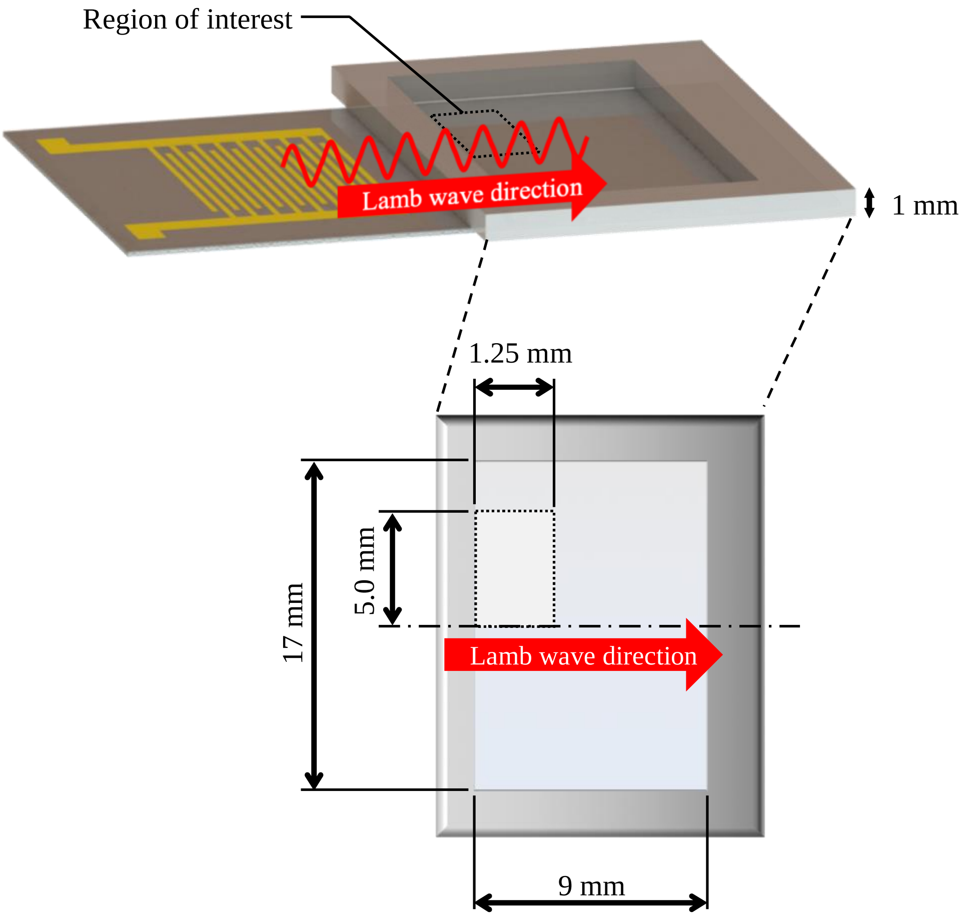

Figure S2 Region of interest on cell culture surface for study of the medium flow and cell detachment. The flow of the media should have a symmetric distribution in this system. Consequently, we have chosen to define our region of interest as the upper half of the entire domain, with the lower boundary represented by the domain and IDT's symmetry axis .

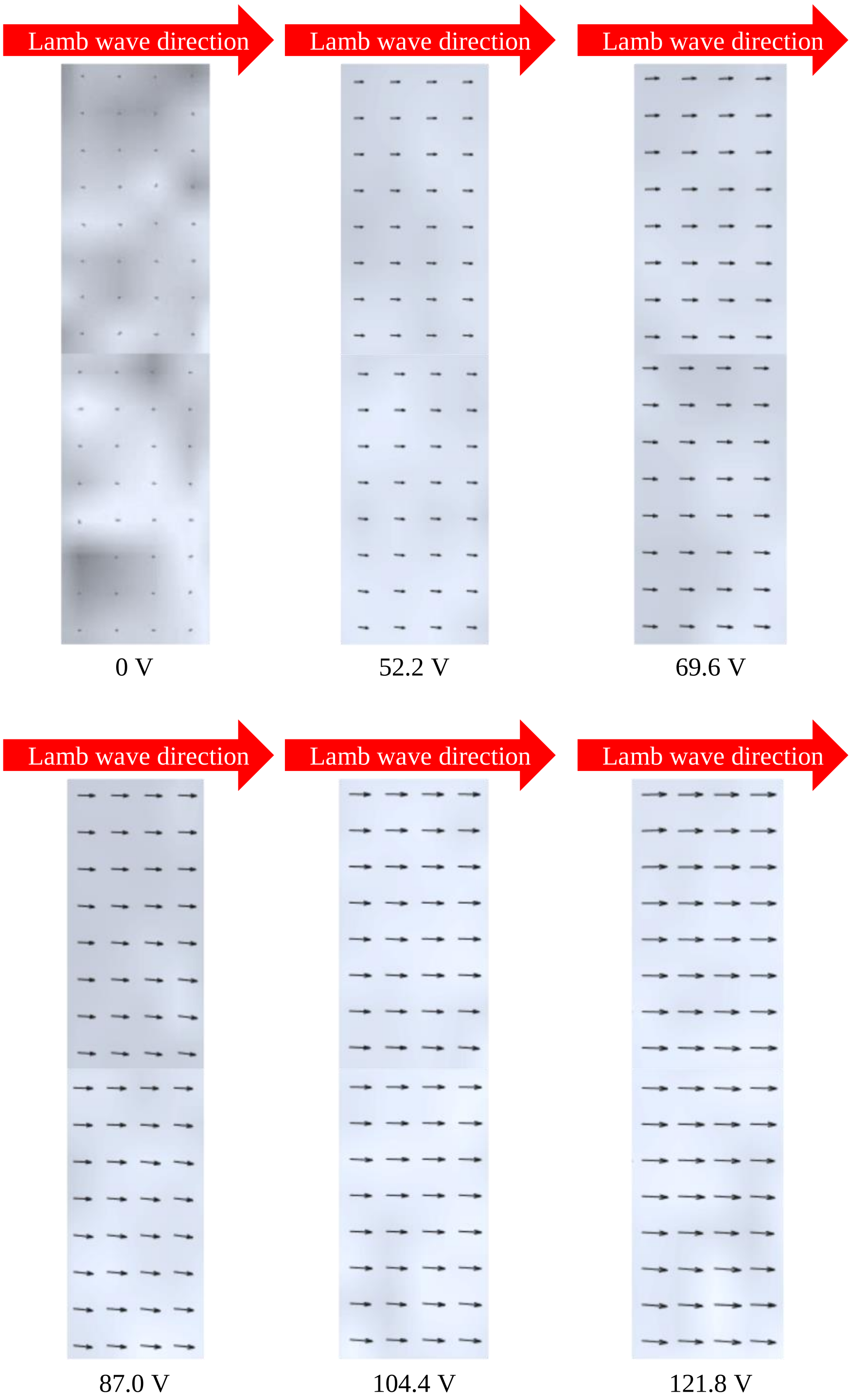

Figure S3 Quiver plots from PIV analysis. The length and direction of arrows represent the velocities of the particles.

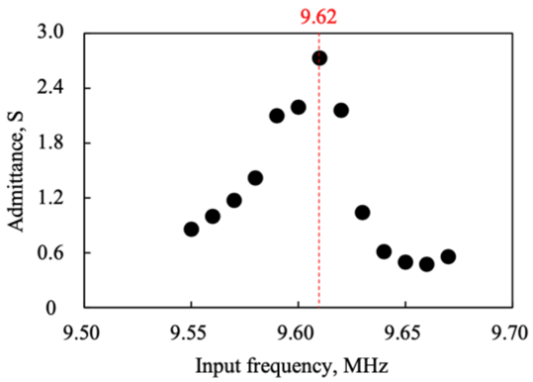

Figure S4 Admittance response of the ultrasound transducer with different input frequencies. Frequency Response Analyzer (FRA5097, NF Corp., Kanagawa, Japan) was used for this measurement.

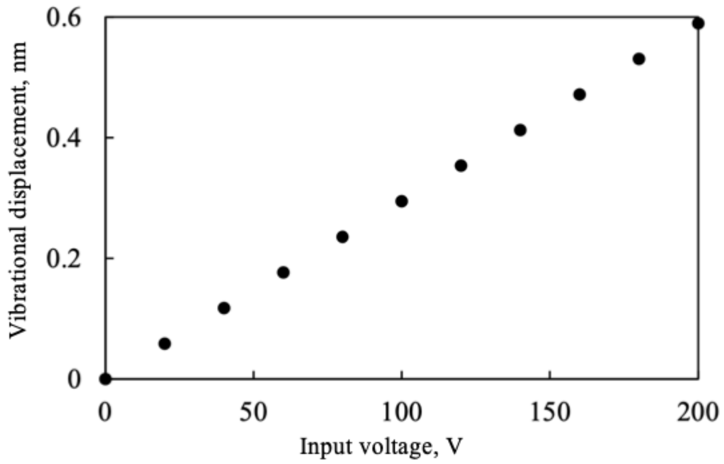

Figure S5 The maximum vibrational displacement calculated by the finite element analysis software. The frequency of 9.62 MHz was employed for input voltages. Maximum vibrational displacement used in this study is 0.5 nm with an input voltage of 173.98 V.

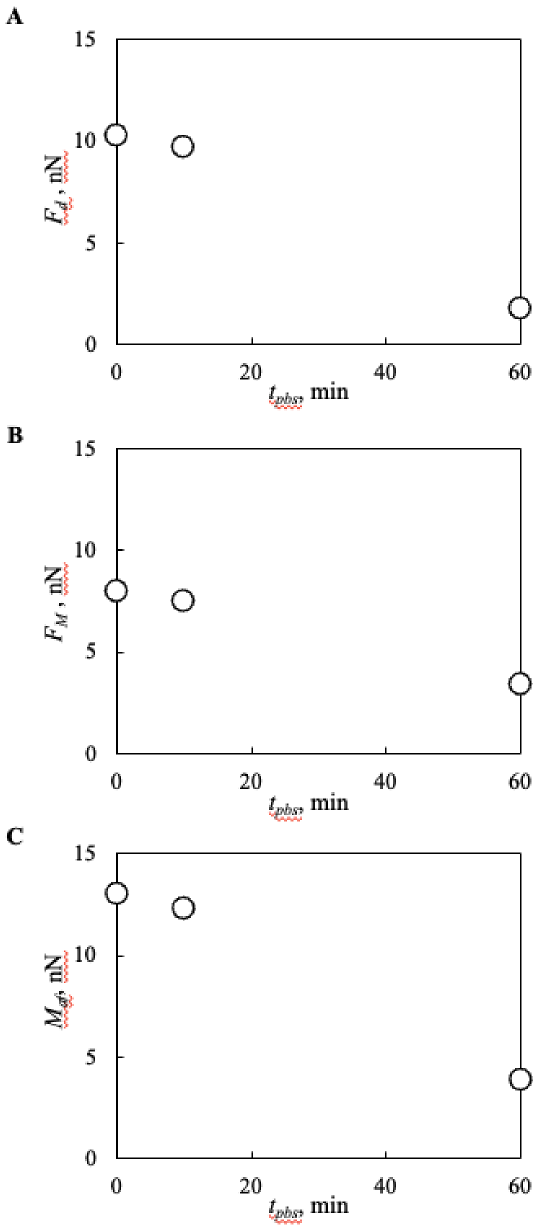

Figure S6 The estimated forces to detach the cells with each incubation time using PBS, t_{pbs}. Vertical axes of (A), (B), and (C) were F_d, F_M, and M_{af}, respectively.

Supplemental Note 1

The vibration amplitude of the Lamb waves should be estimated to evaluate mechanical stimulation. We thus developed a two-dimensional finite element method model of the device using COMSOL 5.5 as shown in Fig. S7. This model consisted of an ultrasound transducer, PDMS wall, and PBS. An electric signal was applied to the IDTs placed on the ultrasound transducer. The dimensions in the computational model were equivalent to those in the experimental setup. The finest mesh size of the computational model was λ/15. Note that λ is the wavelength of the acoustic wave in each domain. Parameters for the lithium niobate domain were adopted from the material library in COMSOL Multiphysics 5.5. The LiNbO3 domain was modeled as piezoelectric material using the "Electrostatics,""Solid Mechanics," and "Piezoelectric Effects" modules. PBS and PDMS were modeled as a linear compressible fluid with acoustic attenuation using the "Pressure Acoustics" module. In terms of electrostatics, domains except for the IDT fingers had electrical insulation boundary conditions. Half of IDT fingers had an electrical potential boundary condition, and the other set of IDT fingers had a ground boundary condition. Pressure release and stress-free conditions were applied to the boundaries of the solid and fluid domains. The bottom and side boundaries of the $LiNbO_3$ domain were set as a low reflection condition due to the absorption of the bulk acoustic wave by the acoustic absorber. At the fluid–solid interfaces, the pressure in the fluid domain was equal to the stress in the solid domain. Wave propagation was governed by the Helmholtz equation.

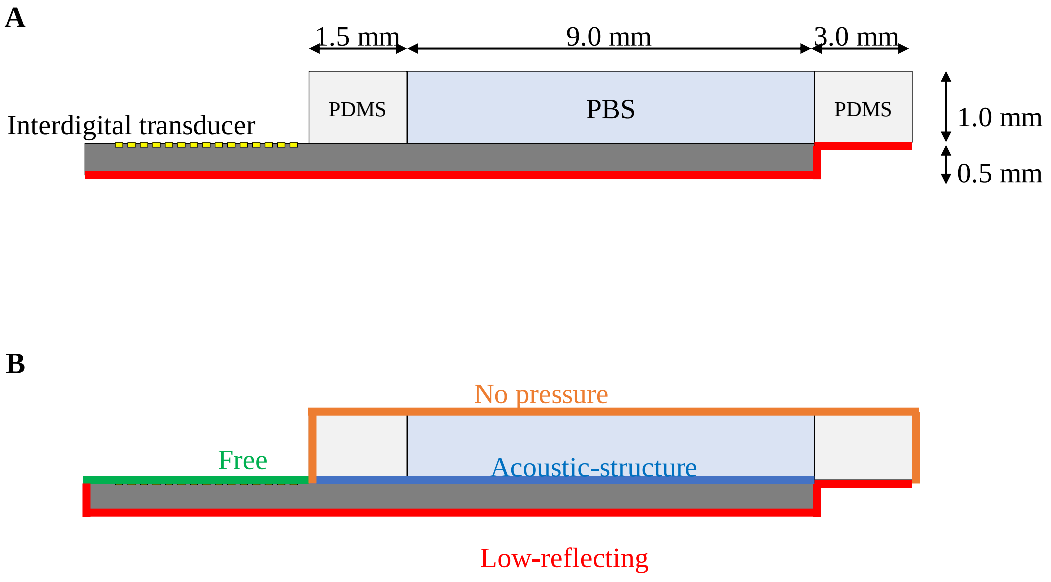

Figure S7 Two-dimensional computational model of the device. The schematic image shows the dimensions (A) and boundary condition (B) of each domain of the computational model (not to scale). The electrical potential was added to the interdigital transducer. The boundary conditions are shown.
